# National Early Warning Scores And COVID-19 Deaths In Care Homes: An Ecological Time Series Study

**DOI:** 10.1093/geroni/igaa057.3517

**Published:** 2020-12-16

**Authors:** Daniel Stow, Robert Barker, Fiona Matthews, Barbara Hanratty

**Affiliations:** 1 Institute of Health and Society, Newcastle upon Tyne, United Kingdom; 2 Newcastle University, Newcastle upon Tyne, United Kingdom; 3 Newcastle University, Newcastle upon Tyne, England, United Kingdom

## Abstract

Tracking COVID-19 infections in the care home population has been challenging, because of the limited availability of testing and varied disease presentation. We consider whether National Early Warning Scores (NEWS/NEWS2) could contribute to COVID-19 surveillance in care homes. We analysed NEWS measurements from care homes in England (December 2019 to May 2020). We estimated pre-COVID (baseline) levels for NEWS and NEWS components using 80th and 20th centile scores for measurements before March 2020. We used time-series to compare the proportion of above-baseline NEWS to area-matched reports of registered deaths in care home residents from the Office for National Statistics We analysed 29,656 anonymised NEWS from 6,464 people in 480 care home units across 46 local authority areas. From March 23rd to May 20th, there were 5,753 deaths (1,532 involving COVID-19, 4,221 other causes) in corresponding geographical areas. A rise in the proportion of above-baseline NEWS was observed from March 16th 2020. The proportion of above-baseline oxygen saturation, respiratory rate and temperature measurements also increased approximately two weeks before peaks in deaths. We conclude that NEWS could contribute to disease surveillance in care homes during the COVID-19 pandemic. Oxygen saturation, respiratory rate and temperature could be prioritised as they appear to signal rise in mortality almost as well as total NEWS. This study reinforces the need to collate data from care homes, to monitor and protect residents’ health. Further work using individual level outcome data is needed to evaluate the role of NEWS in the early detection of resident illness.

